# Can socio-economic differences explain low expectation of health services among HIV patients compared to non-HIV counterparts?

**DOI:** 10.1186/s12889-016-3609-5

**Published:** 2016-09-09

**Authors:** Jing Li, Sawitri Assanangkornchai, Lin Lu, Le Cai, Jing You, Edward B. McNeil, Virasakdi Chongsuvivatwong

**Affiliations:** 1 Epidemiology Unit, Faculty of Medicine, Prince of Songkla University, Hat Yai, Songkhla, 90110 Thailand; 2 Kunming Medical University, Kunming, Yunnan Province China; 3 Yunnan Center for Disease Prevention and Control, Kunming, Yunnan Province China; 4 The First Affiliated Hospital of Kunming Medical University, Kunming, Yunnan Province China

**Keywords:** Expectation, HIV patients, Socio-economic factors, Health system, China

## Abstract

**Background:**

The health service of China has encountered significant challenges due to inequalities in socio-economic determinants of health. HIV patients are known to suffer from social stigma, and may receive inadequate responsiveness from health providers. Before assessing the responsiveness they receive, it is important to know their expectations. We aimed to compare levels of expectation towards the healthcare service among HIV and non-HIV patients with adjustment for socio-economic factors.

**Methods:**

A cross-sectional study was conducted during January and February, 2015 among two consecutive groups of HIV positive and non-HIV patients in two hospitals in Kunming, China. Patients’ expectation towards eight domains of health system responsiveness was measured using 40 vignettes; five per domain. Each vignette was ranked from 1 “very good” to 5 “very bad”, and the responses were summed to obtain a total score for each domain. Differences in total scores were compared between the two groups and adjusted for other factors using multiple linear regression.

**Results:**

The three domains with the highest scores, reflecting high expectation, were prompt attention, basic amenities and choice. Adjusted for other factors, HIV patients had significantly lower levels of expectation in all domains compared to the non-HIV group. Age was associated with the basic amenities domain, with young adults having higher expectations than other age groups. Minority ethnic groups had lower expectation towards dignity, prompt attention and autonomy domains compared to Han ethnicity. Those who lived in a home with 2–4 family members had higher expectations towards confidentiality than those who lived alone.

**Conclusion:**

Patients with HIV have significantly lower levels of expectations even after adjusting for socio-economic factors. Assessment of health system responsiveness based on their judgments above may give biased results toward favorable service quality.

## Background

Patient expectations prior to seeking healthcare services and their perceptions of the care after consuming the service positively affect their satisfaction of the service and confirm or refute their re-visits of the service [1, 2]. Expectations of healthcare systems are proportional to their attractiveness. Patient’s expectations of medical care are linked to the cost of treatment [3], assessments and satisfaction [4, 5]. When the perception of patients towards healthcare meets the expectation of patients [6], a healthcare system will arrive at the perfect level, which appeals to patient-centered medical services [7]. However, there has been little research on the expectation of patients with human immunodeficiency virus infection and acquired immune deficiency syndrome (HIV/AIDS) in comparison to other patients. With the rapid economic development in China, equity of health services faces significant challenges due to a vicious cycle of factors such as inequalities of socio-economic determinants of health [8, 9] and growing dissatisfaction about health system fairness [10, 11] among the public. The high prevalence of HIV/AIDS [12], broad utilization of antiretroviral therapy (ART) and inadequate access to health services [13, 14] combine to create parallel challenges of the HV/AIDS healthcare system. Health systems of China are facing reforms with aims to expand access to more healthcare services and enhance the quality in terms of non-clinical aspects in order to meet the people’s new expectations [15].

According to the WHO framework for assessing the performance of health systems [6], patients’ expectations of healthcare services are categorized into eight domains of health system responsiveness: dignity, confidentiality, communication, autonomy, prompt attention, quality of basic amenities, social support and choice of provider [16]. These domains are related to patient rights, and reflect their expectation of healthcare services according to their perception of healthcare.

In the measurement of expectation, bias due to reporting heterogeneity among survey respondents from different groups with different preferences and cultural norms make cross-cultural comparison of ordinal response categories invalid. A clinical vignette is a short and clear scenario presenting a hypothetical clinical situation, and can resolve this “response-category differential item functioning” [17]. The response of patients to each scenario thereby reveals their perceptions, values, social norms or impressions of clinical events. Such vignettes have been used to assess opinions or preferences across countries, healthcare systems, and specialties [18, 19]. As a marginalized population, HIV/AIDS patients are more vulnerable in healthcare compared to other patients due to the heavy HIV/AIDS stigma and discrimination. However, there is no study focusing on their expectations compared to their counterparts in healthcare setting especially based on vignettes.

Patients’ expectations are affected not only by age [20, 21] and sex, but also by occupation [22, 23], education [23], and income-conventional indicators of socio-economic status (SES). Although different socio-economic indicators have comparable effects on patients’ expectations, a convincing causal relationship between SES indicators and patients’ expectation towards quality of HIV/AIDS healthcare remains to be established.

The presence of socio-economic disparities among HIV patients compared to their non-HIV counterparts may be damaging not only from a human rights perspective but also in sustaining confidence in the system. Identifying the extent of such socio-economic disparities can be the first step in improving the quality of health services and patient satisfaction with services within HIV/AIDS health systems. In this current paper, we aimed to compare levels of expectation of HIV and non-HIV patients in eight domains separately adjusted for different socio-economic factors. The results could be useful for the ongoing healthcare reform process in order to improve the quality of HIV/AIDS care.

## Methods

### Study setting and design

A cross-sectional study was conducted from 1^st^ January 2015 to 15^th^ February 2015. The study was conducted in the infectious departments of two large hospitals: a special infectious hospital and a general hospital in Kunming, the capital city of Yunnan Province, China. The two hospitals have the largest numbers of HIV patients in Kunming. In these hospitals, both HIV/AIDS and non-HIV patients visit the infectious departments. The majority of non-HIV patients have viral hepatitis or other infectious diseases without tuberculosis. All HIV and non-HIV in- and out-patients aged 15 years old or more attending the infectious department of the two study hospitals were eligible to join the study. Patients with tuberculosis were excluded because tuberculosis is one of the most common opportunistic infections of HIV patients. Those who could not communicate in Chinese or were too ill to be interviewed were also excluded. Consecutive sampling was used to recruit study subjects.

### Sample size

Sample size estimation was based on the formula for comparing two population means. We used the confidentiality score to calculate the sample size using the smallest difference between the two groups in a pilot study resulting in the largest sample size. The mean scores (SD) for confidentiality among HIV and non-HIV were 16.77 (3.29) and 17.21 (2.13). With these parameters, the number of subjects required to detect a difference in mean confidentiality scores between two groups, with type one probability of 0.05 and 80 % power, would be 624 per group. To compensate for an estimated 10 % incomplete response rate, 694 were required in each group.

### Development and modification of vignettes

The vignettes were developed by using a standardized protocol from the World Health Survey (WHS) responsiveness module (short version). We firstly selected vignettes for health system responsiveness of Set A to Set D involved in eight domains. Vignettes of Set A focus on two domains: respectful treatment (dignity) and prompt attention, Set B: clear communication and quality of basic amenities, Set C: confidentiality and choice of care provider, and Set D: social support to patient and autonomy. Each set includes ten vignettes, five for each domain. Each vignette simulates patient visits and healthcare provider’s responsiveness to the patient in the relative domain. In each set, ten vignettes of the two domains were mixed in random order.

The vignettes were translated into Chinese and modified by the main researcher to suit the Chinese context. A team of healthcare experts including two chief physicians of infectious departments of the two hospitals, and an expert of HIV/AIDS prevention in the Centre for Disease Control of Yunnan Province, reviewed and finalized the Chinese version of the vignettes. The finalized version was back translated into English and compared with the original version in order to establish the validity of the Chinese version. A focus group discussion consisting of ten non-HIV patients was assembled, and in-depth interviews were conducted with five HIV patients to obtain cultural and contextual relevance. The respondents were asked specific questions in order to determine whether questions were understandable and whether the intent of each question was accurately conveyed. They were also asked to elaborate on the reasons why a particular response category was chosen for a question. According to their suggestions, we modified the vignettes for clearer comprehensibility and cultural suitability. In December of 2014, a pilot study was conducted among 45 HIV and non-HIV patients in both hospitals. It took 60 to 70 min for a patient to complete the questionnaire. The instrument was then shortened to 40 to 60 min duration.

A sample of five vignettes on the dignity domain was as follows:

- [Xiao Zhang] was pregnant and went to the hospital coughing blood. A nurse welcomed her gently and helped her to a private room. A female doctor came to examine her and gave her a clean gown to replace her blood-stained clothes.
- [Xiao Qu] had bad flu. He went to the clinic. The nurse expressed concern about [Xiao Qu]’s cough and called the doctor, who gave [Xiao Qu] a full chest examination behind a large screen that hid him from the view of other patients.
- [Xiao Ting] went to a crowded clinic. At first, no-one greeted her but after waiting for 5 min a nurse called her to the examination area where she was examined behind a small screen that mostly hid her from the other patients.
- [Wang Li] took her baby for a vaccination. The nurse said hello and but did not ask for [Wang Li’s] or the baby’s name. The nurse also examined [Wang Li] and made her remove her shirt in the waiting room.
- [Luo Ping] has AIDS. When he goes to his health center the nurses do not talk to him and deliberately ignore him. During examinations, his clothes are removed and he is made to wait, half-naked in the waiting room.

All vignettes for the dignity domain were followed by the question: “How would you rate his/her experience of being greeted and talked to respectfully?” The participants were then instructed to answer this question using a Likert rating scale ranging from 1 representing “very good” to 5 representing “very bad” and 3 representing “moderate”. Similar questions were asked for each domain with the same rating scale used to obtain participant’s responses.

### Study variables and measures

Dependent variables were the total scores of the eight domains as measured by five vignettes per domain. All five responses were summed to obtain a total score for each domain, with a possible range of 5 to 25, where higher scores indicate higher expectation towards that domain. Demographic variables, measured by a self-reported questionnaire, included age, gender, ethnicity, religion, place of residence, marital status, family size, education, occupation, and household income. For comparability with other studies, age was arbitrarily grouped into three categories: (i) 40 years old or less (young adults); (ii) 41 to 60 years old (middle-aged); (iii) more than 60 years old (elderly). The nine ethnic groups were classified into two categories: Han and other ethnicity. Place of residence was classified as either rural or urban based on their insurance type. Family size was grouped into 3 categories: (i) single; (ii) 2–4; (iii) 5 or more family members. SES factors included education, occupation, and household income per month. Education was grouped into four levels: (i) primary school or less; (ii) junior high school; (iii) senior high school, and (iv) university or more. Occupation was grouped into four categories: (i) government-employed; (ii) enterprise-employed; (iii) self-employed; (iv) unemployed. Household income was categorized into five levels according to distribution of household income by place of residence in China: (i) 800RMB or less; (ii) 801 ~ 2000RMB; (iii) 2001 ~ 5000RMB; (iv) 5001 ~ 8000RMB; (v) 8001RMB or more.

### Data analysis

Comparison of sample characteristics between HIV positive and non-HIV patients was performed using Chi-square goodness-of-fit tests for categorical variables, and t-tests for continuous variables. Comparisons of mean scores for the eight domains were done using t-tests or analysis of variance (ANOVA) as appropriate. Multiple linear regression models were conducted separately for each domain to assess their independent association with demographic variables and SES factors. Variables having a p-value less than 0.05 were considered as significant. All analyses were performed using R language and environment.

## Results

Two consecutive groups containing 696 HIV and 699 non-HIV patients were included in the study. The response rate was 87 % and 66 % among HIV and non-HIV patients, respectively.

### Demographics and socio-economic status

Table 1 shows the distribution of demographic and socio-economic variables. The majority of patients were male, of Han ethnicity, married or cohabiting, and employed. Most reported having no religious affiliation. About half achieved a junior high school level of education and had a monthly household income of 5000 RMB or less and living in a family of size 2–4 members. Both groups were closely matched on gender; however, HIV positive patients were more likely to belong to a minority ethnicity, have a religious affiliation, live in rural areas, have a higher education level, be separated, divorced or widowed, have a lower household income, live with fewer family members and be self-employed.

**Table 1:** Distribution of socio-demographic variables

		Total sample		HIV patients		Non-HIV patients		p*
		(*n* = 1395)		(*n* = 696)		(*n* = 699)		
		n	%	n	%	n	%	
Age								
	<=40	803	57.6	422	60.6	381	54.5	0.048
	41-60	502	36.0	236	33.9	266	38.1	
	> = 61	90	6.5	38	5.5	52	7.4	
Gender								
	Female	549	39.4	270	38.8	279	39.9	0.709
	Male	846	60.6	426	61.2	420	60.1	
Ethnic group								
	Han	1094	78.4	504	72.4	590	84.4	<0.001
	Other	301	21.6	192	27.6	109	15.6	
Religious affiliation								
	No	1149	82.4	522	75.0	627	89.7	<0.001
	Yes	246	17.6	174	25.0	72	10.3	
Place of residence								
	Rural	758	54.3	490	70.4	268	38.3	<0.001
	Urban	637	45.7	206	29.6	431	61.7	
Marriage								
	Single	282	20.2	159	22.8	123	17.6	<0.001
	Married/Cohabiting	935	67.0	383	55.0	552	79.0	
	Separated/Divorced/Widowed	178	12.8	154	22.1	24	3.4	
Family size								
	1	58	4.2	52	7.5	6	0.9	<0.001
	2–4	1030	73.8	535	76.9	495	70.8	
	> = 5	307	22	109	15.7	198	28.3	
Education								
	<=Primary school	306	21.9	144	20.7	162	23.2	<0.001
	Junior high school	668	47.9	296	42.5	371	53.3	
	Senior high school	311	22.3	158	22.7	152	21.8	
	> = University	110	7.9	98	14.1	11	1.6	
Occupation								
	Government-employed	129	9.2	62	8.9	67	9.6	<0.001
	Enterprise-employed	499	35.8	256	36.8	243	34.8	
	Self-employed	213	15.3	176	25.3	37	5.3	
	Unemployed	554	39.7	202	29.0	352	50.4	
Household income (Yuan)								
	<800	244	17.5	163	23.4	81	11.6	<0.001
	801–2000	322	23.1	177	25.4	145	20.8	
	2001–5000	417	29.9	184	26.4	233	33.3	
	5001–8000	239	17.1	88	12.6	151	21.6	
	> = 8001	173	12.4	84	12.1	89	12.7	

*: All *p* values in the column were from Chi-squared tests

### Differences in eight domains between HIV and non-HIV patients

Table 2 presents mean scores of eight domains of patients’ expectation of healthcare between HIV and non-HIV patients, based on the vignettes. Of all domains, HIV patients had significantly lower mean expectation scores than non-HIV patients.

**Table 2:** Distributions of patients’ expectation scores based on vignettes

	Total	HIV patients	Non-HIV patients	p*
	(*n* = 1395)	(*n* = 696)	(*n* = 699)	
Dignity				
	14.0 (2.5)	13.9 (2.7)	14.2 (2.2)	0.024
Prompt attention				
	15.9 (2.8)	14.9 (3.0)	17.0 (2.2)	<0.001
Communication				
	14.4 (2.2)	14.1 (2.5)	14.6 (1.7)	<0.001
Basic amenities				
	15.5 (2.2)	15.2 (2.6)	15.8 (1.7)	<0.001
Confidentiality				
	17.0 (2.7)	16.8 (3.3)	17.2 (2.0)	0.004
Choice				
	15.7 (2.6)	15.3 (3.0)	16.2 (2.0)	<0.001
Social support				
	14.1 (2.2)	13.8 (2.5)	14.4 (1.9)	<0.001
Autonomy				
	14.3 (2.2)	14.2 (2.6)	14.5 (1.8)	0.004

*: *p* values from independent *t*-test

### Multivariate analyses

After adjustment for demographic and socio-economic variables, HIV status remained significantly associated with lower expectations of all health system domains (Table 3). Age was significantly associated with basic amenities, with young adults having a higher expectation. Compared to Han people, minority ethnic groups had lower expectations towards dignity, prompt attention and autonomy. Those who lived in a family containing 2–4 members had a higher expectation than those who lived alone.

**Table 3:** Multiple linear regression of patients’ expectation based on vignettes among eight domains

	Dignity		Prompt attention		Communication		Basic amenities		Confidentiality		Choice		Social support		Autonomy	
	Coeff. (95 % CI)	*p*	Coeff. (95 % CI)	*p*	Coeff.(95 % CI)	*p*	Coeff. (95 % CI)	*p*	Coeff. (95 % CI)	*p*	Coeff. (95 % CI)	*p*	Coeff. (95 % CI)	*p*	Coeff. (95 % CI)	*p*
HIV status: Non-HIV vs. HIV		0.027		<0.001		<0.001		<0.001		0.003		<0.001		<0.001		0.0046
	0.252 (−0.009,0.513)		2.106 (1.826,2.386)		0.532 (0.302,0.762)		0.587 (0.354,0.821)		0.397 (0.102,0.692)		0.928 (0.662,1.195)		0.543 (0.308,0.778)		0.288 (0.052,0.524)	
Age: ref. = 16–40								0.0177								
41–60							−0.35 (−0.598,-0.102)									
61–85							−0.29 (−0.78,0.201)									
Ethnic group: other vs. Han		0.0398		0.0178												0.0046
	−0.333 (−0.65,-0.016)		−0.412 (−0.752,-0.071)												−0.415 (−0.701,-0.128)	
Family size: ref. = 1										0.0389						
2–4									0.912 (0.179,1.644)							
> =5									0.74 (−0.045,1.525)							

**Fig. 1:**
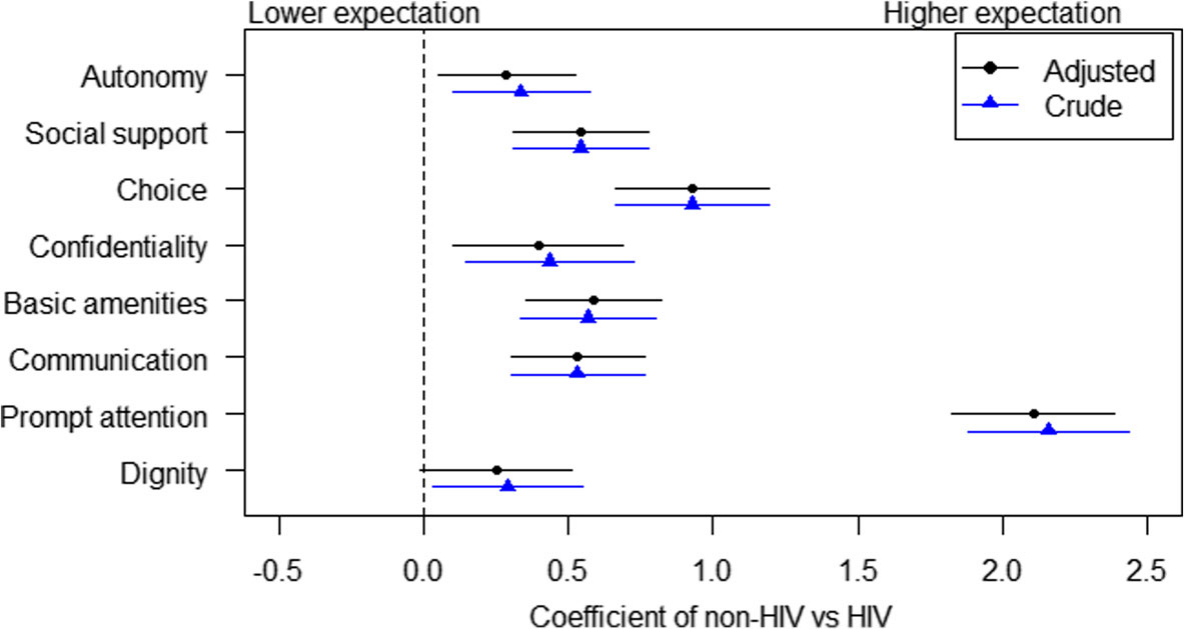
Differences in health service expectation between HIV and non-HIV patients

Figure 1 compares the crude and adjusted coefficients from the linear regression models among each domain, reflecting the differences in expectation scores between HIV positive and non-HIV patients. Prompt attention had the highest coefficient reflecting a relatively higher expectation by non-HIV patients. Non-HIV patients also had higher expectations towards basic amenities, choice of provider, confidentiality, communication, autonomy, social support and dignity.

## Discussion

HIV patients had lower expectation scores in all health system domains even after adjustment by demographic and socio-economic factors, reflecting lower expectations of the healthcare system. Compared to non-HIV patients, they were slightly younger, belonged to a minority ethnic group, more religious, more educated, self-employed, more likely to be single or separated and had lower household incomes. Thus, on top of having a lower SES, HIV positive patients in this study were further oppressed by their own HIV status.

Not many studies have focused on differences in expectations of healthcare system between HIV and non-HIV patients across socio-economic status. One study showed that patient’s satisfaction with nursing care was associated with younger age, being male, being non-white and having HIV [24]. However, that study did not find an independent effect of being HIV positive after adjustment for SES factors.

Among the eight health system domains, prompt attention was found to have the highest difference of expectation between HIV positive and non-HIV patients, and the low expectation of prompt attention by HIV patients suggested that there is a shortage of human resources and a lack of an efficient mechanism to uniformly cooperate in HIV/AIDS care. Additionally, quality of basic amenities is linked to health facilities. One study confirmed that this domain is not strongly correlated with clinical quality, and depends on different hospitals in terms of productivity based on instrumental variables [25]. The lower expectation of HIV patients towards basic amenities reflects their helplessness about dissatisfaction with designated hospitals because of not only limited medical resources but also “logistic choices” to hospitals or providers. Another study considered consulting the same healthcare provider to be a source of comfort in provider-patient relationships. However, the comforting affection from seeing the same provider is on the premise that patients have free choice rights [26, 27]. The monitoring and evaluation system of China cannot equally share the whole medical resources, and there is a lack of effective operational mechanisms to respond timely to the patient’s needs. Under this system, the free choice rights of HIV patents have not been taken into account.

The lower HIV patient expectation in confidentiality can sometimes create a dilemma for health professionals or family members because there is a fine line between safeguarding their privacy and the need to inform other people about their illness. Some studies documented the benefit to patients, especially those with HIV/AIDS, based on human-rights, but others hold the opposite view [28–30]. Besides these, some suggested to identify boundaries of confidentiality [31]. The majority of people living with HIV/AIDS (PLWHA) often avoid naming themselves in public, to their neighbors, and even sometimes to their own family members. As a marginalized population, they are more vulnerable because of the heavy HIV/AIDS stigma [32, 33], especially discrimination by healthcare providers. When disclosing their HIV status, the majority of providers in non-appointed hospitals will refuse to examine and treat them and transfer them to special HIV unit. During their care, there was little dignity [34] given to them because of a lack of effective communication, and lack of prompt attention and respect for individual autonomy [35] such as self-decisions and meaningful participation. Thus, elimination of stigma is an important goal in the struggle against HIV/AIDS for subsequent HIV testing and counselling, and adherence to ART. Additionally, confidentiality, choice of provider, dignity and clarity of communication are deserved rights of HIV positive patients. Adopting a human rights-based approach towards care of HIV/AIDS patients can be very helpful to improve access to HIV prevention, care and treatment.

In terms of social support, HIV patients had a lower expectation compared to their counterparts. Most HIV positive patients expect that they will stay by themselves in hospital, but other patients expect care and contact from their family and friends. The fact that HIV patients abandon their right of access to family and community support may be a consequence of social stigma. Other evidence has shown that decision-making interventions can improve quality of healthcare [36]. This suggests that empowerment of HIV patients within the healthcare system will strengthen quality of healthcare.

Policies in China such as “Four Frees and One Care” has had a great success on expanding the coverage of prevention of mother-to-child transmission and ART. Another policy called “HIV/AIDS regulation” first highlighted human rights’ protection in early 2006 [37]. However, the effects of empowering these marginalized people in China is lacking. Evidence has shown that empowerment of PLWHA has resulted in policy changes, especially regarding access to free ART. For example, Thailand’s response to HIV/AIDS is considered one of the best success stories due to civil society groups as networks at different levels promoted the efficient coordination of activities [38]. Free access to ART has brought massive relief, restoring people’s health and enabling them to care for families, providing hope for the future and allowing PLWHA to participate in community activities [39]. In addition, success of Treatment Action Campaign in South Africa, a powerful force in converting donor perceptions of universal access to treatment into a moral imperative, led to policy changes for a global impact in 2004. However, free access to ART cannot replace empowerment of PLWHA in which human rights and fundamental freedoms can be realized.

### Limitations

There are some limitations in our study, which should be acknowledged. Firstly, we could not involve patients-family-friends relationships during the decision-making process of seeking healthcare services in our vignette because there are various roles that family or friends play in Chinese culture. Moreover, selection bias was unavoidable since those who did not seek healthcare services or did not know their HIV status were not entered into our study.

## Conclusion

HIV positivity was associated with a lower expectation of the health system, which could not be explained by any socio-economic indicators. Assessment of health system responsiveness based on HIV patient’s perception may give biased results toward quality of HIV prevention, care and treatment services, had existing of expectation on care been ignored. In addition, a human rights-based approach to HIV/AIDS patients should be implemented.
